# Supplementary material for: Vertical ocean heat redistribution sustaining sea-ice concentration trends in the Ross Sea
Source: Nat Commun. 2017 Aug 15;8:258. doi: 10.1038/s41467-017-00347-4 (PMC5557847; doi:10.1038/s41467-017-00347-4)
Supplement: Supplementary file 1 — Supplementary Information [file 41467_2017_347_MOESM1_ESM.pdf]

Description of Supplementary Files

File Name: Supplementary Information
Description: Supplementary Figures and Supplementary References

File Name: Peer Review File

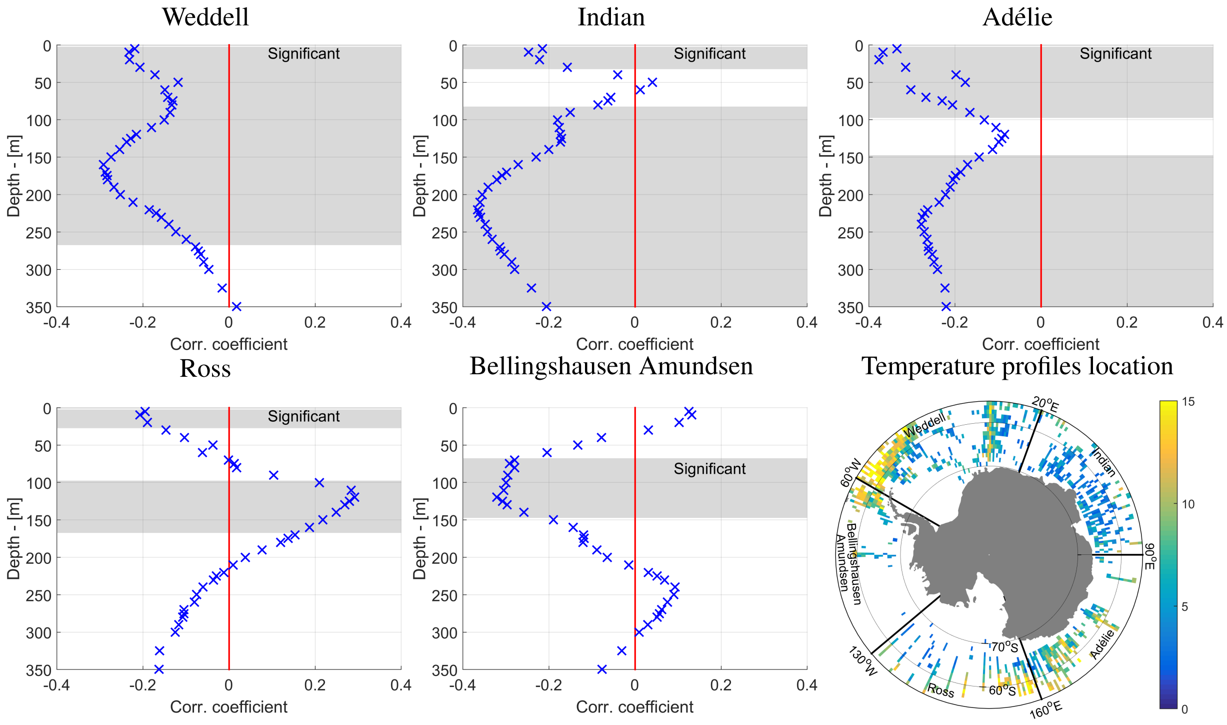

Supplementary Figure 1|**Observation-based correlation between 1979-2014 trends in ice concentration and ocean temperature.** Correlation coefficients between ice concentration trends and ocean temperature trends as a function of depth from observations (OSISAF[2] and BLUELink Ocean Archive[3]) are displayed for each sector of the Southern Ocean. Grey areas show the depths at which the correlations are significant at the 95% confidence level. The additional map in the last panel provides the spatial distribution of the ocean temperature profiles used to compute the correlations. The color represents the number of years containing at least one record in a given grid cell over 1979-2014.

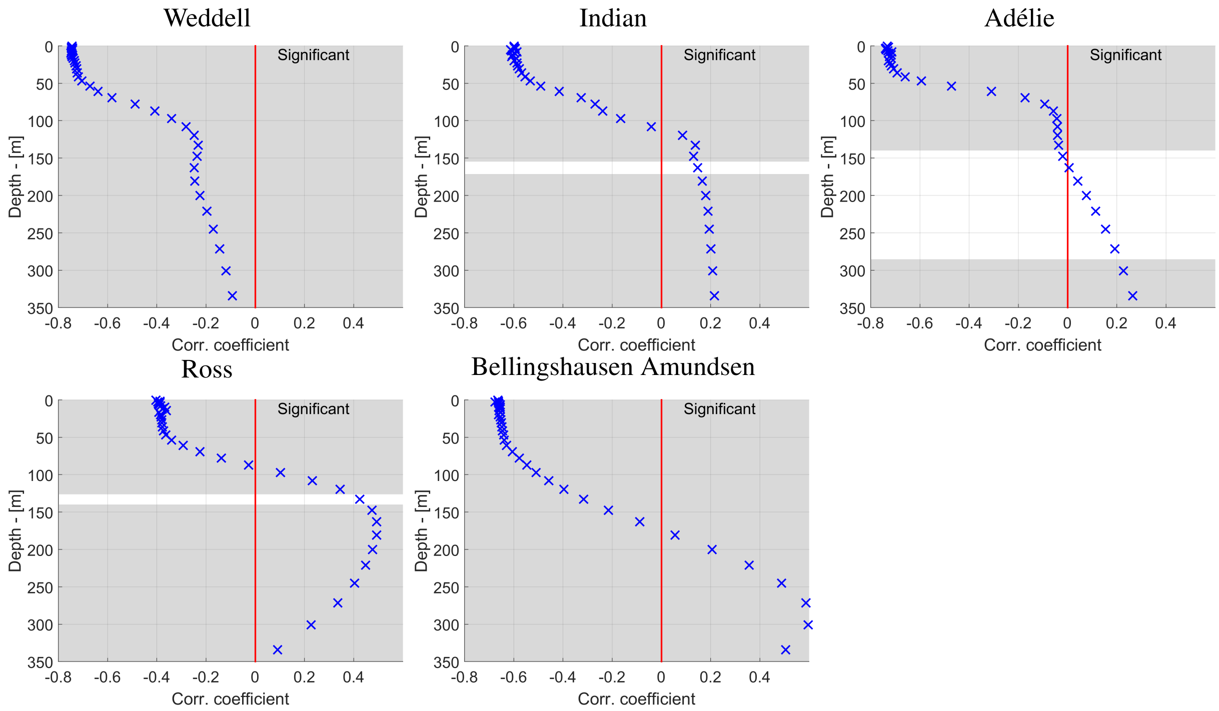

Supplementary Figure 2|**Model-based correlation between 1979-2013 trends in ice concentration and ocean temperature.** Correlation coefficients between ice concentration and ocean temperature trends as a function of depth from NEMO-LIM3.6, for each sector of the Southern Ocean. Grey areas show the depths at which the correlations are significant at the 95% confidence level.

a

b

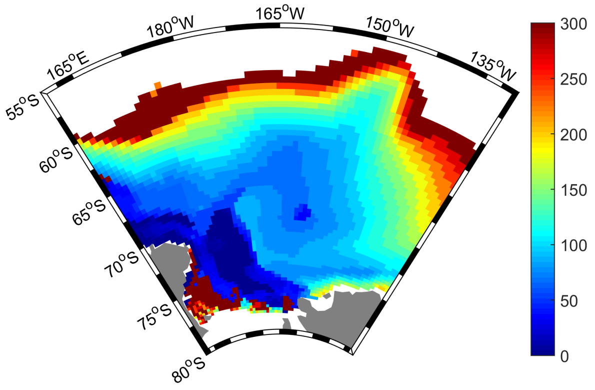

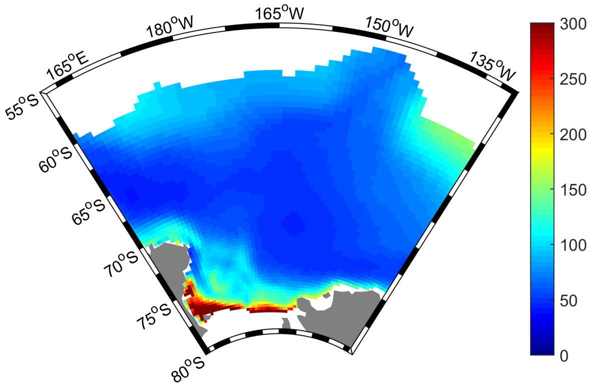

Supplementary Figure 3|**Depth of the maximum ocean temperature trends and mixed-layer depth in the Ross Sea.** (a) Depth of the maximum ocean temperature trend (1979-2013) in the first 300 m below the surface and (b) mean mixed-layer depth over 1979-2013 in the seasonally ice-covered region of the Ross Sea, from model data.

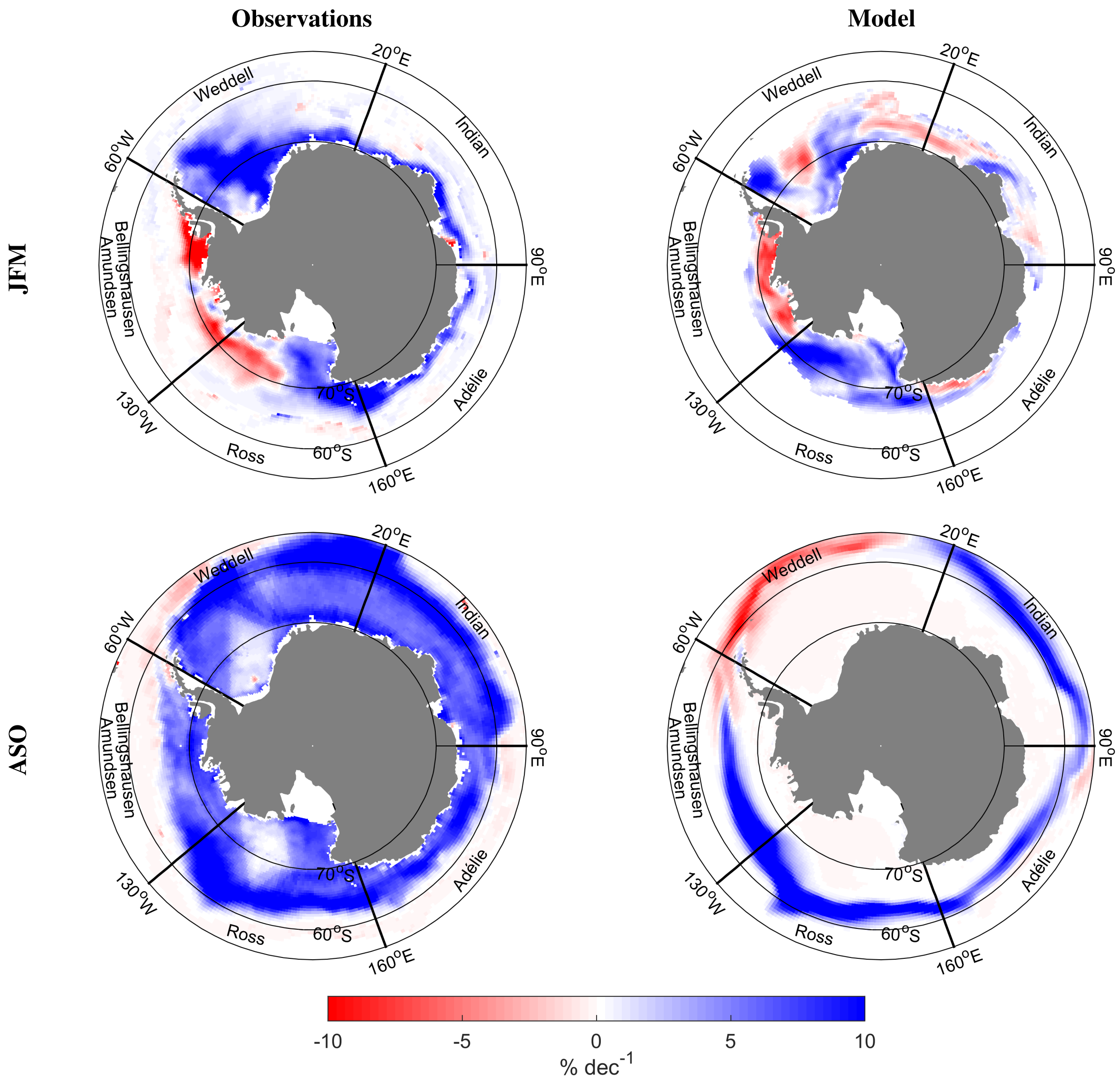

Supplementary Figure 4|**1979-2014 (observations) and 1979-2013 (model) seasonal trends in sea-ice concentration in the Southern Ocean.** JFM and ASO stand for January-February-March and August-September-October mean trends in ice concentration, respectively. The ASO observation-based trends exhibit a clear swath-shaped anomaly over the Weddell and Ross Seas related to the satellite data coverage. This is due to a local inconsistency in the raw OSISAF data in the years 1979-1984 and at this time of the year, but no impact can be seen on the trends based on annual mean ice concentrations analyzed in this study.

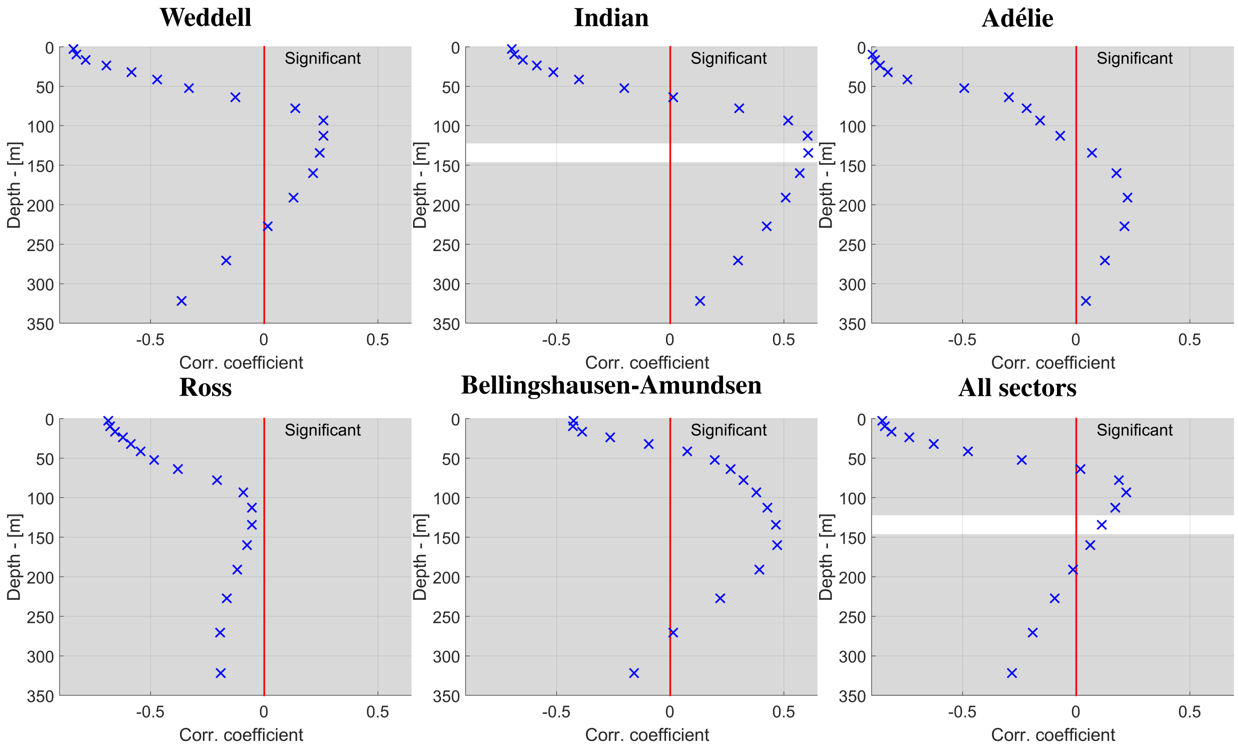

Supplementary Figure 5|**Correlation between 1952-1978 trends in ice concentration and ocean temperature at depth.** Correlation coefficients are displayed for a model simulation[4] (described in Methods) in each sector of the Southern Ocean and for all sectors together. Grey areas show the depths at which the correlations are significant at the 95% confidence level.

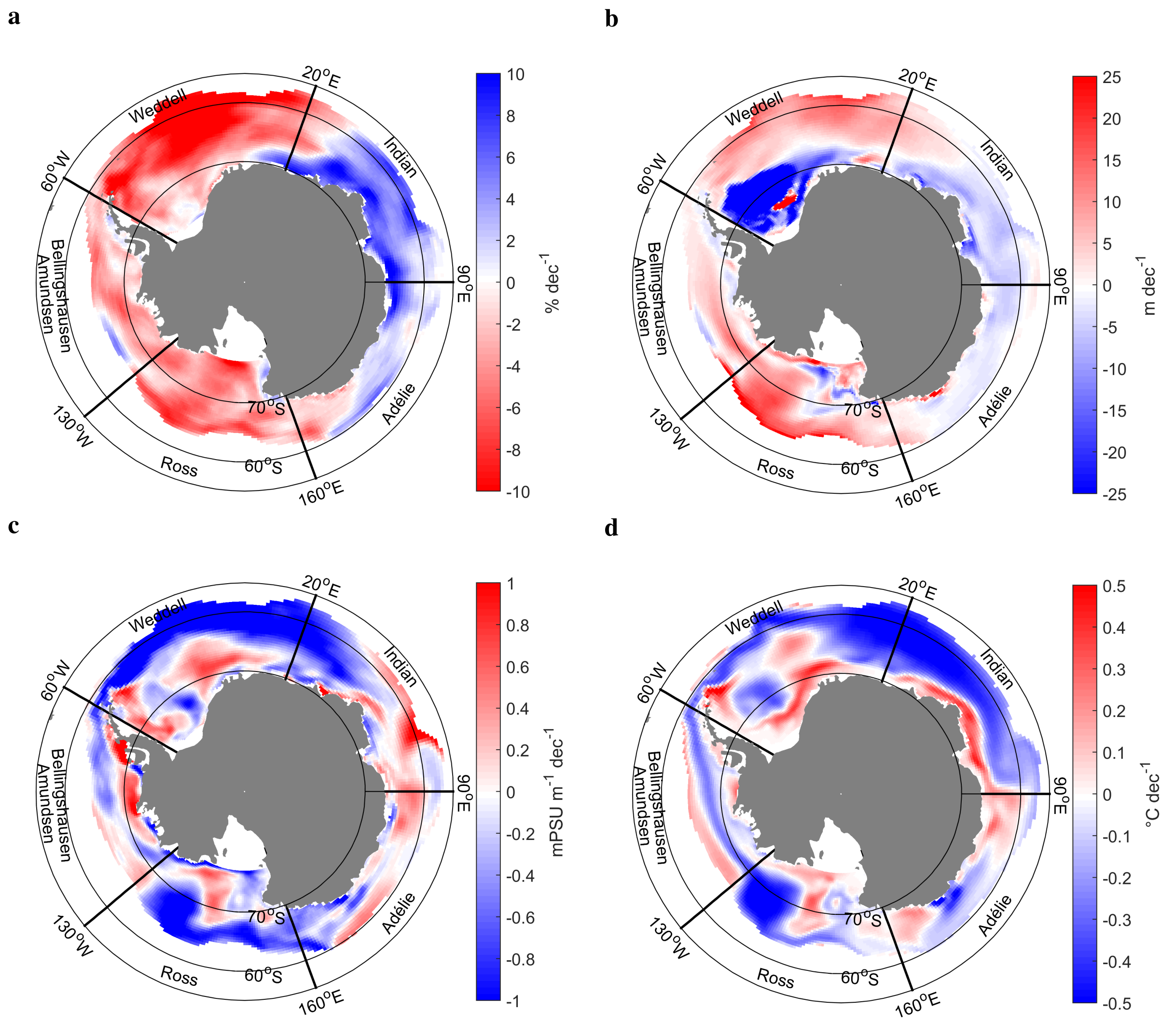

Supplementary Figure 6|**1952-1978 trends in sea-ice concentration and upper ocean properties over the Antarctic ice-covered region.** Trends in (a) sea-ice concentration, (b) mixed-layer depth, (c) salinity gradient in the top 150 m and (d) ocean temperature at 150 m below the surface are displayed for a model simulation[4] (see Methods).

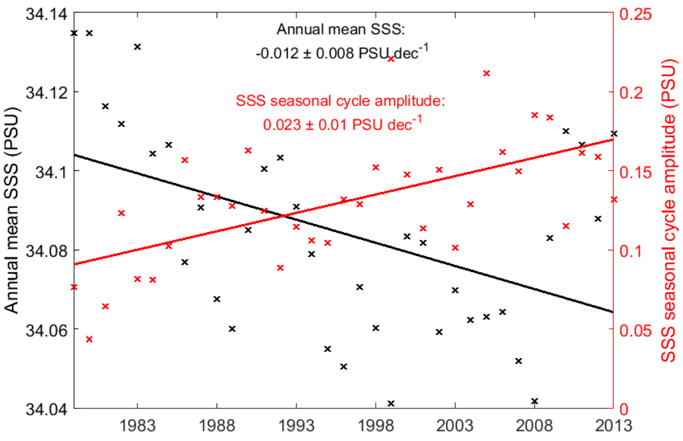

Supplementary Figure 7|**Model-based annual time series and trends (1979-2013) in sea surface salinity (SSS) and amplitude of the SSS seasonal cycle, averaged over the Ross Sea areas covered by sea ice in winter and north of -65° South.**

Supplementary References

1. Schmidtko, S., Heywood, K. J., Thompson, A. F. & Aoki, S. Multidecadal warming of Antarctic waters. *Science* **346**, 1227–1231 (2014).

2. EUMETSAT Ocean and Sea Ice Satellite Application Facility. Global sea ice concentration reprocessing dataset 1978-2015 (v1.2, 2015), [Online: http://osisaf.met.no]. Norwegian and Danish Meteorological Institutes .

3. Ridgway, K. R., Dunn, J. R. & Wilkin, J. L. Ocean interpolation by four-dimensional weighted least squares-application to the waters around Australasia. *Journal of atmospheric and oceanic technology* **19**, 1357–1375 (2002).

4. Barthélemy, A., Fichefet, T., Goosse, H. & Madec, G. Modeling the interplay between sea ice formation and the oceanic mixed layer: Limitations of simple brine rejection parameterizations. *Ocean Modelling* **86**, 141–152 (2015).
